# Reply to Dr. J. Morgan Howe

**Published:** 1901-01

**Authors:** C. N. Peirce

**Affiliations:** Philadelphia


### Domestic Correspondence.

### REPLY TO DR. J. MORGAN HOWE.

PHILADELPHIA, November 20, 1900.

TO THE EDITOR:

SIR,—In the November issue of the INTERNATIONAL DENTAL JOURNAL I have read with pleasure an article on "Professional Dignity," by Dr. J. Morgan Howe. The keen sense of propriety and appropriate ethical conduct which Dr. Howe in his daily life represents makes him always an interesting writer and speaker.

It is possible, however, that he has taken rather a pessimistic view of the profession, and of college graduates, when he refers to Dental Parlors and their promoters as representatives of college graduates. It is true, however, that the commercial spirit is an important factor in dental journalism as well as in dental practice, but it is not necessarily true that this spirit or desire for profit or gain is antagonistic to the ethics of either medicine or dentistry. Nor is it true that such journalism is retarding "professional progress;" nor is it true that "trade channels," largely influential in publishing dental as well as medical literature, are inconsistent with true professional dignity.

It is, however, unfortunately true that what we are pleased to term "independent journalism" has a life and death struggle, and that the publication of expensively illustrated articles must seek such sources of publicity as can be fostered by the profits of trade.

Trade is not necessarily unethical; it is certainly not in that degraded condition that the truly professional man must say, with holy horror, "'Touch me not' for I am 'holier than thou.'"

The writer has been for many years quite, yes, very conversant with men interested in trade, and, without expecting to be in harmony with every act, there is much to be admired in results that have been achieved through trade. Unselfish and self-sacrificing have transactions often been; temptations have been resisted that would have made individuals independently wealthy had they yielded. It would not be difficult to bring to the doctor's mind efforts on the part of men that have been exhausting, expensive, and most perplexing, yet persistent and untiring labor has been pursued which has liberated hundreds, if not thousands, of others from burdens and impositions, that would not only have been onerous but exasperating, and all done in a perfectly ethical spirit.

Does not Dr. Howe realize that but for the protection of associated effort he, with many others, might now be paying unjust royalties on many delicate and artistic operations. And yet upon the instigators of this protection he casts a slur, with an expressed desire to cut loose in the matter of ethics from those interested in, or conducting, such associations. He can certainly broaden his mantle of charity without being unethical.

The doctor has trodden upon sensitive ground when he speaks of colleges using preparations of which nothing is known save the name of the maker; he of course alludes to alloys and zinc cements. The writer must make his protest. Nothing has ever been used in the clinics of the school with which he has been connected that was not definitely familiar to the professor in the special department, and every professor teaching the practical branches in the good dental schools is certainly thoroughly conversant with all the ingredients in both of these compositions. He also knows that slight variations in the quantity of the several ingredients, and subsequent treatment, may, indeed does, produce varying results, and that the annealing process, which is now so universally applied to alloys, has its beneficial influence on the manipulative process and final results. All of this knowledge in well-conducted schools is carefully given to the student by the professor having charge of that practical branch.

The doctor greatly laments the commercial control of dental literature. If it will be of any comfort, let me remind him of the condition with which he is certainly familiar,—that is, that medical literature is equally under trade control.

While the human body has to be nourished and protected from thermal changes, nothing will be so sensitive as the profits or emoluments of labor, mental and physical. But this profit ought all to be acquired without violation of ethical conduct, and most heartily will the writer commend any and every effort the doctor can make towards elevating the moral tone of societies, practitioners, and colleges.

C. N. Peirce.

